# Risk Factors Associated with Endoscopic Intervention in Pediatric Patients Presenting with Foreign Body Ingestion to the Emergency Department

**DOI:** 10.3390/children12101344

**Published:** 2025-10-07

**Authors:** Young-hoon Byun, Ji Eun Kim, So Hyun Paek, Min-Jung Kim, Soo Hyun Park, Ho-Young Song, Jin Hee Kim, Sung-Ha Kim, Jae Hyun Kwon

**Affiliations:** 1 Department of Emergency Medicine, CHA Bundang Medical Center, CHA University School of Medicine, Seongnam 13496, Republic of Korea; byunyoun84@chamc.co.kr (Y.-h.B.); hyun21400@chamc.co.kr (S.H.P.); mjtear8287@chamc.co.kr (M.-J.K.); suas11@cha.ac.kr (S.H.P.); shyped85@chamc.co.kr (H.-Y.S.); heartfeltdoctor@chamc.co.kr (J.H.K.); paulie1018@chamc.co.kr (S.-H.K.); 2 Department of Emergency Medicine, Dong-A University Hospital, Busan 49201, Republic of Korea; amcfsapple@dau.ac.kr

**Keywords:** foreign body ingestion, esophagogastroduodenography, pediatric emergency department

## Abstract

**Background/Objectives**: Pediatric foreign body (FB) ingestion is a common clinical problem that frequently necessitates time-sensitive decisions regarding esophagogastroduodenoscopy (EGDS). Although established high-risk criteria guide the indication for EGDS, coins—despite their high prevalence—are not uniformly classified as high-risk FBs. In this study, we aimed to delineate epidemiology and endoscopic outcomes and to identify predictors of EGDS. **Methods**: We retrospectively reviewed cases of children younger than 15 years presenting to an urban emergency department (ED) with suspected or confirmed FB ingestion between 2014 and 2020. After applying exclusion criteria, 757 patients remained for analysis. Data abstracted included demographic characteristics, presenting symptoms, type and location of FB, ED length of stay (EDLOS), and whether EGDS was performed along with its outcomes. Multivariable logistic regression was used to identify predictors of EGDS, including age group, sex, symptom presence, established high-risk criteria, and type of FB (coin, button battery, magnet). **Results**: Among 757 children (median age 17.0 months; 54.0% male), 55.2% were asymptomatic. EGDS was performed in 47 of the 757 children (6.2%), with a success rate of 74.5% (35/47). Among EGDS cases, the most common foreign bodies were coins (29.8%), button batteries (27.7%), and magnets (17.0%). In multivariable models, established high-risk criteria were the dominant determinant of EGDS (adjusted OR ≈ 179.4; 95% CI, 29.9–1075.8; *p* < 0.001). Compared with button batteries, coin ingestion was independently associated with EGDS (adjusted OR ≈ 7.26; 95% CI, 1.07–49.31; *p* = 0.042). **Conclusions**: Established high-risk criteria were the primary determinant of EGDS, and coin ingestion showed a possible independent association with EGDS; these findings suggest that coin ingestion may warrant consideration as a potential high-risk factor when determining whether endoscopy is indicated.

## 1. Introduction

Pediatric foreign body (FB) ingestion is a common and clinically important condition that occurs predominantly from 6 months to 3 years of age and presents a major indication for pediatric esophagogastroduodenoscopy [1,2,3].

Pediatric foreign body ingestion occurs more frequently in males [4] and is predominantly accidental [5]. Evaluation and management should integrate the type and size of the ingested object, the circumstances and elapsed time since ingestion, and the child’s age and clinical condition [5,6]. However, history and physical examination alone are often insufficient to confirm FB ingestion [7], and many children initially present to the emergency department (ED) either asymptomatic or with nonspecific findings such as irritability or feeding refusal [8]. When present, symptoms include vomiting, drooling, choking, chest pain, and feeding refusal [9]. An esophageal FB may compress the posterior trachea wall or larynx, provoking cough, wheezing, stridor, or a choking sensation [10]. Hematemesis and abdominal pain/tenderness can indicate upper or lower gastrointestinal (GI) complications, whereas abdominal rigidity or rebound tenderness may indicate perforation [6,11].

Most foreign bodies (FBs) pass through the gastrointestinal (GI) tract uneventfully [6], with those in the distal GI tract typically being expelled spontaneously [6]. The most common sites of impaction include the upper esophageal sphincter, mid-esophagus, lower esophageal sphincter, pylorus, an ileocecal valve [12]. In children, the mean GI transit time is approximately 3.6 days [13]; persistence of an FB beyond this interval, or the presence of red-flag clinical features including drooling, choking, dyspnea or hematemesis, warrants consideration of endoscopic removal or surgical intervention [14,15,16].

Complications are more frequently observed when either patient-related or foreign body (FB)-related risk are present [6]. High-risk FBs include elongated, sharp or pointed objects, button batteries, and small magnets [6,17]. Key patient-related risk factors that have been reported include functional or anatomical abnormalities, such as esophageal or duodenal strictures and prior gastrointestinal surgery, and underlying conditions such as dysphagia, achalasia, esophagitis, and Down syndrome [18,19,20,21,22,23,24].

Coins are the most frequently ingested foreign bodies in children [25]. Although larger-diameter coins, particularly when ingested by infants and young children, are associated with an increased risk of esophageal impaction or impaired passage through the pylorus [26], coins have not traditionally been classified as high-risk foreign bodies. This study reviewed the medical records of pediatric patients presenting with foreign body ingestion to a single emergency center, with the aim of characterizing the epidemiology and clinical features and identifying risk factors for endoscopic intervention (EGDS), with particular emphasis on larger-diameter coins.

## 2. Methods

### 2.1. Study Design and Participants

This retrospective observational study was conducted in the emergency department of a single urban resident-training hospital. Electronic medical records of 923 patients younger than 15 years with suspected or confirmed FB ingestion between 1 January 2014 and 31 December 2020 were reviewed. A complete EGDS was performed in all patients undergoing endoscopic intervention—including those with esophageal FBs to exclude additional FBs in the stomach or duodenum.

### 2.2. Eligibility Criteria and Definitions

Eligible participants included all children under 15 years of age who presented to the ED with suspected or confirmed FB ingestion. High-risk FBs were defined by the presence of one or more of the following seven criteria: (1) sharp or wide/elongated objects (≥2.5 cm in width or ≥6 cm in length), (2) multiple FBs, (3) button batteries, (4) evidence of perforation, (5) a coin at the cricopharyngeus muscle level, (6) airway compromise, and (7) presence ≥24 h (Table A1). Age groups were categorized as infants (<12 months), toddlers (12–23 months), preschooler (24–71 months), and school-aged children (72–168 months).

### 2.3. Data Collection

Employing a standardized abstraction form, we collected data on demographics (age, sex); presence and type of initial symptoms categorized by system involvement (gastrointestinal, respiratory, neurologic); FB characteristics (type, material, size); anatomic location confirmed by radiographic imaging or EGDS (esophagus, stomach, small bowel, or unknown); ED length of stay (EDLOS); and whether EGDS was performed, along with its outcome (success or failure). In FB type classification, the category ‘unknown’ was assigned when FB’s material composition was indeterminate, FB was not witnessed by caregivers, or no FB was identified on diagnostic imaging.

For EGDS cases, we additionally collected the location of FB at EGDS, type-specific proportions and success rates of endoscopy, actual measured size of FB, reasons for failed removal (found but not removed, not found, or managed with observation) and procedure-related complications. In the overall cohort, we examined age-specific distributions of three major FB types (coins, button batteries, magnets); in EGDS cases, we assessed type-specific anatomic distributions.

### 2.4. Outcome Measures

The primary outcome was to identify risk factors for endoscopic intervention in pediatric foreign body ingestion—including established high-risk FB categories, age group, sex, presence and type of symptoms, and type of FB—with particular emphasis on whether coin ingestion should be classified as high risk. Secondary outcomes included: (i) description of epidemiology and clinical characteristics of the entire cohort (age distribution, presenting symptoms, type and location of FB, EDLOS); (ii) characterization of the EGDS subgroup (location, type, size of FB) and the success rate of endoscopic removal; and (iii) comparisons of the characteristics of EGDS utilization and EDLOS between patients with high-risk versus non-high-risk FBs.

### 2.5. Statistical Analysis

All statistical analyses were performed using R software (version 4.3.0, R Foundation for Statistical Computing, Vienna, Austria).

Baseline demographic and clinical characteristics are summarized using descriptive statistics. Categorical variables are expressed as frequencies and percentages, and continuous variables as means with standard deviations or medians with interquartile ranges, depending on distribution. Between-group comparisons were conducted using the chi-square test or Fisher’s exact test for categorical variables, and Student’s *t*-test or Mann–Whitney U test for continuous variables, according to normality assessed by the Shapiro–Wilk test.

To identify independent predictors of EGDS, a multivariable logistic regression model was constructed. Given the limited sample size, only the three most frequent foreign body types among patients who underwent EGDS (coin, button battery, and magnet) were included as categorical predictors, along with age group, sex, symptom presence, and high-risk classification. This restriction was applied to ensure model stability and to avoid unreliable estimates arising from sparsely populated categories. Adjusted odds ratios (aORs) with 95% confidence intervals (CIs) were calculated, and a two-tailed *p*-value of <0.05 was considered statistically significant.

### 2.6. Follow-Up and Ethics

At discharge, families were instructed to seek outpatient reassessment or return to the ED as indicated; admission was arranged when clinically indicated. The study received institutional review board approval, and informed consent was waived due to the retrospective design.

## 3. Results

**Figure 1:**
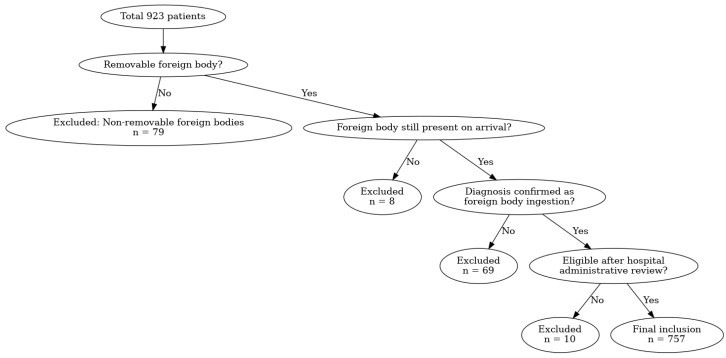
Flowchart of patient inclusion and exclusion.

During the study period, 923 ED visits presented with suspected or confirmed FB ingestion. Exclusions were as follows: 79 with FBs not requiring removal, 8 who presented after FB removal elsewhere, 69 ultimately not diagnosed with FB ingestion, and 10 who were not evaluated due to personal or institutional reasons. The final analytic cohort consisted of 757 patients (Figure 1).

Among 757 children, males accounted for 409 (54.0%) and females for 348 (46.0%). The mean age was 27.2 ± 24.3 months; the median age was 17.0 months [IQR 10.0–38.0 Age-group distribution was 235 infants (31.0%), 241 toddlers (31.8%), 230 preschoolers (30.4%), and 51 school-aged children (6.7%). Symptoms were absent in 418 patients (55.2%), 186 (24.6%) had gastrointestinal symptoms, 217 (28.7%) had respiratory symptoms, and 8 (1.1%) had neurologic symptoms (categories not mutually exclusive). Patients with symptoms falling within two or more categories were assigned to all relevant symptom categories. The distribution of FB types was as follows: 126 plastic (16.6%), 91 vinyl/sticker (12.0%), 92 metal (12.2%), 73 food (9.6%), 59 magnetic (7.8%), 37 soft/synthetic (4.9%), 62 battery (8.2%), 61 coins (8.1%), 97 others (12.8%), and 59 unknown (7.8%). The “others” category comprised glass, stone, seed, animal bone, wood, and paper; “unknown” referred to cases in which the ingested object could not be identified. The median EDLOS was 40.0 min [IQR 27.0–64.0] (Table 1).

EGDS was performed in 47 of 757 patients (6.2%), with an overall success rate of 74.5% (35/47). In the subgroup, the mean age was 36.8 ± 32.2 months, and the median was 20.0 months [IQR 13.0–47.5]. Age-group distribution was 7 infants (14.9%), 17 toddlers (36.2%), 15 preschoolers (31.9%), and 8 school-aged children (17.0%). 19 patients (40.4%) were symptomatic and 28 (59.6%) asymptomatic. FB types included 14 coins (29.8%), 13 batteries (27.7%), 8 magnets (17.0%), 3 metal objects (6.4%), 2 plastic items (4.3%), 3 soft synthetic materials (6.4%), 3 other objects (6.4%), and 1 food (2.1%). At the time of EGDS, FBs were located in the esophagus (20, 42.6%), in the stomach (20, 42.6%), or in the small bowel (1, 2.1%), with 6 cases not found (12.8%). The mean measured size was 21.2 ± 7.3 mm, and the median was 22.0 mm [IQR 17.5–26.0] (missing *n* = 4; 8.5%) (Table 2) Of 12 failed attempts, 5 were categorized as found but not removed, 6 as not found, and 1 as found but judged unnecessary for removal and managed with observation. No major procedure-related complications were reported, and all patients were discharged safely.

The proportion of males was higher in the high-risk group than in the non-high-risk group (63/92 [68.5%] vs. 346/665 [52.0%]; *p* = 0.004). Age was higher in the high-risk group (mean 41.6 ± 32.4 months; median 35.5 [IQR 13.8–57.0]) than in the non-high-risk group (mean 25.7 ± 22.7 months; median 16.0 [IQR 8.0–34.0]) (*p* < 0.001). Age-group distribution showed a significantly larger proportion of school-aged children relative to infants in the high-risk group (*p* < 0.001). Differences in symptom categories were not statistically significant (gastrointestinal *p* = 0.672, respiratory *p* = 0.061, neurologic *p* = 0.607). With respect to FB type, the high-risk group had higher proportions of battery (28.3%) and coin (29.3%) (*p* < 0.001). EGDS was performed more frequently in the high-risk group (37.0%) than in the non-high-risk group (2.0%; *p* < 0.001). EDLOS was longer in the high-risk group (74.0 min [IQR 37.0–184.5]) than in the non-high-risk group (37.0 min [IQR 26.0–58.0]; *p* < 0.001) (Table 3).

**Figure 2:**
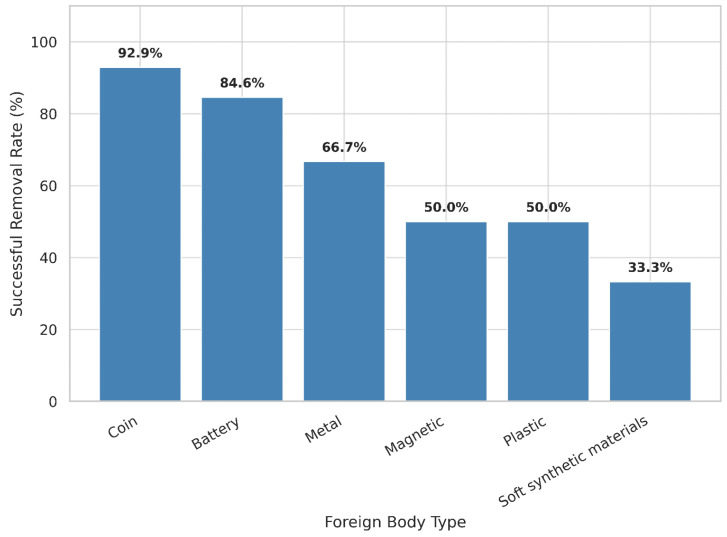
Successful Removal Rates by FB Type in Esophagogastroduodenoscopy Cases.

Among the 47 EGDS cases, the overall success rate of removal was 74.5%. Success rates varied substantially according to FB type: coins 92.9%, button batteries 84.6%, metal 66.7%, plastic 50.0%, magnets 42.9%, and soft synthetic materials 33.3%. Although not depicted in the figure, food-related FBs had 0% (*n* = 2), whereas others (glass, stone, seed, animal bone, wood, etc.) had 33.3% (*n* = 24) (Figure 2).

**Figure 3:**
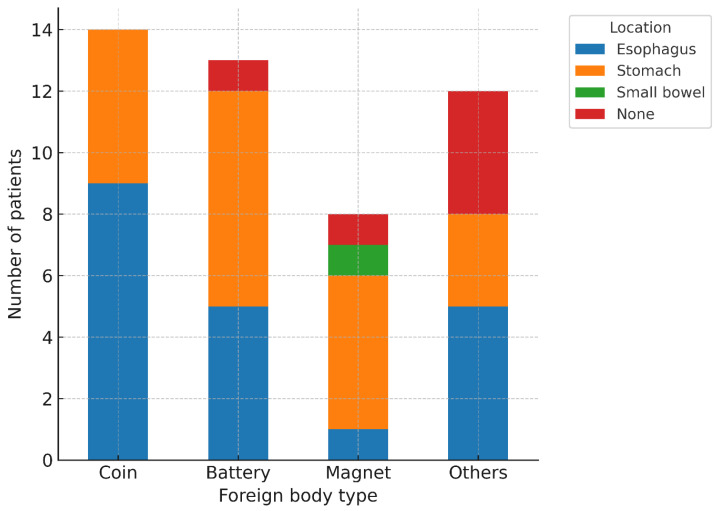
Anatomical Distribution of FBs by Type in Esophagogastroduodenoscopy Cases.

In the 47 EGDS cases, FBs were located in the esophagus (20, 42.6%), the stomach (20, 42.6%), the small bowel (1, 2.1%), or were undetermined (6, 12.8%). By type, coins were located in the esophagus (9, 64.3%) and the stomach (5, 35.7%); button batteries in the esophagus (5, 38.5%), the stomach (7, 53.8%), and undetermined (1, 7.7%); magnets in the esophagus (1, 12.5%), the stomach (5, 62.5%), the small bowel (1, 12.5%), and undetermined (1, 12.5%); and other objects in the esophagus (5, 41.7%), the stomach (3, 25.0%), and undetermined (4, 33.3%) (Figure 3). Overall, FBs were predominantly located in the esophagus and the stomach, with only rare small-bowel involvement (2.1%).

**Figure 4:**
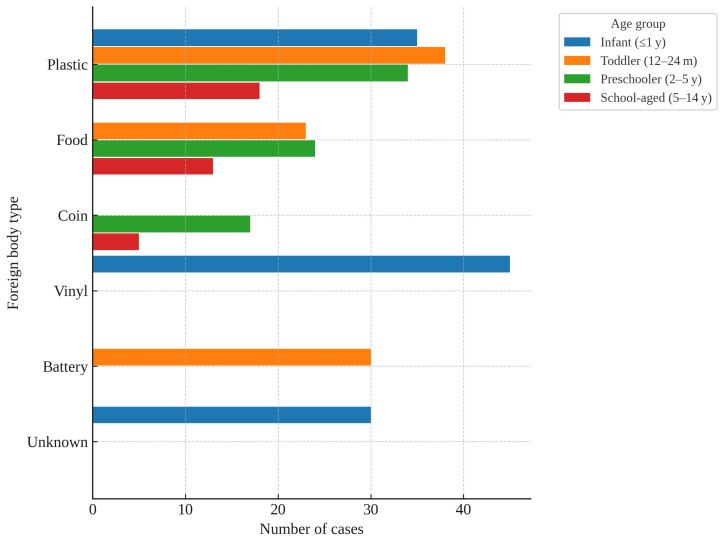
Age-specific Distribution of the Three Most Common Foreign Body Types.

The distribution of FB types varied markedly across age groups. In infants (≤1 year), vinyl (45, 39.8%), plastic (34, 30.1%), and unknown objects (30, 26.5%) were the most common. In toddlers (12–24 months), plastic (36, 28.6%), batteries (30, 23.8%), and food (22, 17.5%) predominated. In preschoolers (2–5 years), plastic (35, 35.4%), food (23, 23.2%), and coins (17, 17.2%) were frequent. In school-aged children (5–14 years), plastic (15, 34.1%), food (14, 31.8%), and coins (6, 13.6%) were most frequent (Figure 4). Plastic and food were consistently common across all age groups, whereas batteries were particularly prominent in toddlers, and coins in preschool- and school-aged children.

In multivariable logistic regression, high-risk FBs were most strongly associated with EGDS (aOR ≈ 179.4, 95% CI 29.9–1075.8; *p* < 0.001). Coin ingestion showed a significant association with EGDS compared with battery ingestion (aOR ≈ 7.26, 95% CI 1.07–49.31; *p* = 0.042) (Table 4). Age group, sex, symptom, and magnets were not independently associated with EGDS.

## 4. Discussion

The predominance of coins in pediatric FB ingestion [25,27], together with evidence that some mid or distal esophageal coins may progress to the stomach within a short interval whereas proximal esophageal coins rarely advance spontaneously [28], aligns with our finding that EGDS was most frequently performed for coins and button batteries and that FBs were predominantly localized to the esophagus and stomach. These findings highlight that coin ingestion, although common, should not be underestimated, as it showed a possible association with the need for EGDS, even compared with button batteries. Accordingly, coin ingestion may warrant consideration as a potential high-risk factor in clinical decision-making for pediatric FB management.

Because 20–38% of patients with an esophageal FB may be asymptomatic [10,29], and chest pain, fatigue, or fever can indicate esophageal perforation [11], early endoscopic evaluation should be prioritized according to established high-risk FB criteria, even when a substantial proportion of patients are asymptomatic. Complications increase when patient- or FB-related risk factors coexist [6] and can range from acute deterioration to delayed sequelae [30,31]. Within this context, EGDS is essential: it enables removal of the FB, identification of additional FBs, and assessment of mucosal injury [6]. Moreover, even with negative radiographic findings, persistent symptoms may be considered an indication for diagnostic and therapeutic EGDS [32,33]. The timing of EGDS should be determined after consideration of multiple factors, including patient age, body weight, time interval since last oral intake, elapsed time since ingestion, and characteristics of FB (type, size, shape, and location) [34]. For button batteries in the esophagus, endoscopic removal within 2 h is recommended [35], and sharp, elongated, or bulky FBs should be removed within 24 h [36].

The key objective of this study is to identify novel risk factors for EGDS. Although coins are among the most commonly ingested FBs in children, they are not consistently classified as high-risk FBs in current guidelines. In our multivariable logistic regression analysis, the established high-risk criteria remained the strongest determinant; however, coin ingestion also showed a possible independent association with EGDS when compared with button batteries. This finding suggests that, contrary to the prevailing perception that button batteries represent the highest risk, coin ingestion may also represent a potential indicator for endoscopic intervention and warrant inclusion in high-risk criteria in future guideline revisions. Accordingly, at triage, coin ingestion might be taken into account as an additional weighted criterion alongside the established high-risk criteria.

Additionally, the probability of successful endoscopic FB removal varied according to the type and location of foreign body. Coins, button batteries, and magnets were predominantly located in the esophagus and stomach, facilitating endoscopic access. In contrast, soft synthetic and food-related FBs demonstrated a low success rate for endoscopic removal (33.3%; Figure 1), likely due to the poor visualization of retained food materials, distal migration of FB beyond the reach of EGDS, technical difficulty of secure capture and frag-mentation during the procedure. Clinically, these findings suggest the potential role of a systematic preprocedural evaluation of FB type and characteristics and support the consideration of early surgical consultation when endoscopic removal is anticipated to be technically challenging.

In the analysis of FB location by type (Figure 2), coins and button batteries were predominantly found in the upper gastrointestinal tract—particularly in the esophagus and stomach—whereas magnets and other objects were more widely distributed throughout the gastrointestinal tract. These findings suggest that the urgency of EGDS may need to be determined in relation to the suspected type of FB; for FBs with a high likelihood of esophageal impaction, expedited endoscopy may be warranted.

In our study, the distribution of FB types also differed markedly across age groups (Figure 3). Plastic and food-related FBs were consistently common across all ages, likely reflecting their ready availability in daily life. By contrast, button batteries were particularly prevalent among toddlers, underscoring the vulnerability of this age group to hazardous household items, whereas coins were more prominent among preschool- and school-aged children—patterns plausibly attributable to age-related increases in access to small metal objects in household and school environments. Such age-specific distribution patterns may aid decision-making in the ED by informing both the likelihood of FB type and the urgency with which EGDS should be performed.

Taken together, these patterns of anatomical and age-specific distribution support the consideration of FB type and patient age in risk stratification when assessing the urgency and necessity of EGDS.

This study has several limitations. First, as a single-center retrospective analysis, its external validity may be limited. The threshold for EGDS referral may have differed by the requesting clinician in emergency department and by time of presentation (nighttime or weekend hours), which could have resulted in differences in EDLOS and proportion of patients undergoing EGDS. Because the time from FB ingestion to ED presentation was ascertained from caregiver reports, it may be imprecise and prone to misclassification. In addition, due to the retrospective nature of our study, several clinical variables could not be incorporated. Factors such as patients’ comorbidities and prior interventions before transfer may have influenced the likelihood and success of EGDS but were not captured in our dataset. The relatively small number of patients who underwent EGDS (*n* = 47) left some categories sparsely populated in the logistic regression, resulting in wide 95% confidence intervals and precluding precise estimation of true effect size. With sparse data, coefficients can be unstable and either inflated or attenuated severely; accordingly, the odds ratio for high-risk classification exhibited a particularly wide CI. These findings should be interpreted with caution, and larger prospective cohorts are warranted to obtain more precise and robust estimates and to confirm these associations.

Due to limited sample size, certain FB categories (e.g., plastic, food, and other less frequent objects) were excluded from the regression analyses to avoid unstable estimates. Consequently, determinants of EGDS for these less common FBs could not be assessed, which may limit the generalizability of our findings. Despite these limitations, our study provides novel evidence that coin ingestion is independently associated with the need for EGDS, underscoring the importance of considering coins as high-risk foreign bodies in pediatric patients.

## 5. Conclusions

In pediatric foreign-body ingestion, established high-risk criteria were the primary determinant of EGDS, and coin ingestion showed a possible independent association with the procedure. These findings suggest that coin ingestion may warrant consideration as a potential risk factor when determining the need for EGDS. Large-scale prospective multicenter studies are needed to clarify determinants for less common FB types.

## Figures and Tables

**Table 1:** Characteristics of Pediatric Patients with Foreign Body Ingestion.

**Gender**	^1^
Male	409 (54.0)
Female	348 (46.0)
**Age, months**	
Mean, SD	27.2 ± 24.3
Median IQR	17.0 [10.0–38.0]
**Age group**	
Infant (<12 months)	235 (31.0)
Toddler (12–23 months)	241 (31.8)
Preschooler (2–5 years)	230 (30.4)
School-aged (6–14 years)	51 (6.7)
**Symptoms**	
Gastrointestinal	186 (24.6)
(abdominal pain, nausea, vomiting, diarrhea, oral bleeding)	
Respiratory	217 (28.7)
(sore throat, cough, drooling, cyanosis, wheezing, chest pain)	
Neurologic	8 (1.1)
(seizure, irritability)	
None	418 (55.2)
**Foreign body type**	
Plastic	126 (16.6)
Metal	92 (12.2)
Vinyl/Sticker	91 (12.0)
Food	73 (9.6)
Battery	62 (8.2)
Coin	61 (8.1)
Magnetic	59 (7.8)
Soft synthetic materials	37 (4.9)
Others	97 (12.8)
Unknown	59 (7.8)
**EDLOS, minutes**	
Median, IQR	40.0 [27.0–64.0]

^1^ Values are presented as number (%) unless otherwise indicated. Age is expressed as mean ± standard deviation (SD) and median [interquartile range, IQR]. EDLOS = emergency department length of stay.

**Table 2:** Clinical Characteristics of Patients who Underwent Esophagogastroduodenoscopy.

**Gender**	^1^
Male	31 (66.0)
Female	16 (34.0)
**Age, months**	
Mean, SD	36.8 ± 32.2
Median IQR	20.0 [13.0–47.5]
**Age group**	
Infant (<12 months)	7 (14.9)
Toddler (12–23 months)	17 (36.2)
Preschooler (2–5 years)	15 (31.9)
School-aged (6–14 years)	8 (17.0)
**Symptoms**	
Present	19 (40.4)
None	28 (59.6)
**Foreign body type**	
Coin	14 (29.8)
Battery	13 (27.7)
Magnetic	8 (17.0)
Metal	3 (6.4)
Plastic	2 (4.3)
Soft synthetic materials	3 (6.4)
Others	3 (6.4)
Food	1 (2.1)
**Foreign body location**	
Esophagus	20 (42.6)
Stomach	20 (42.6)
Small bowel	1 (2.1)
None	6 (12.8)
**Foreign body size, mm**	
Mean, SD	21.2 ± 7.3
Median, IQR	22.0 [17.5–26.0]
**Successful removal**	35 (74.5)
**High-risk ingestion**	34 (72.3)

^1^ Values are presented as number (%) unless otherwise indicated. Age and foreign body size are expressed as mean ± standard deviation (SD) and median [interquartile range, IQR].

**Table 3:** Comparison of Clinical Characteristics Between High-risk vs. Non high-risk Groups.

*	Non High-Risk	High-Risk	*p*-Value
**Gender**			
Male	346 (52.0)	63 (68.5)	
Female	319 (48.0)	29 (31.5)	0.004
**Age, months**			
Mean, SD	25.2 ± 22.2	41.6 ± 32.4	<0.001
Median, IQR	16.0 [10.0–35.0]	35.5 [13.8–57.0]	<0.001
**Age group**			
Infant (<12 months)	225 (33.8)	10 (10.9)	
Toddler (12–23 months)	212 (31.9)	29 (31.5)	
Preschooler (2–5 years)	173 (26.0)	32 (34.8)	
School-aged (6–14 years)	55 (8.3)	21 (22.8)	<0.001
**Symptom**			
Gastrointestinal	149 (22.4)	23 (25.0)	0.672
Respiratory	164 (24.7)	14 (15.2)	0.061
Neurologic	8 (1.2)	0 (0.0)	0.607
None	406 (61.1)	61 (66.3)	
**Foreign body type**			
Plastic	123 (18.5)	3 (3.3)	
Vinyl/Sticker	91 (13.7)	0 (0.0)	
Metal	78 (11.7)	14 (15.2)	
Food	71 (10.7)	2 (2.2)	
Magnetic	46 (6.9)	13 (14.1)	
Soft synthetic materials	39 (5.9)	2 (2.2)	
Battery	36 (5.4)	26 (28.3)	
Coin	34 (5.1)	27 (29.3)	
Others	88 (13.2)	5 (5.4)	
Unknown	59 (8.9)	0 (0.0)	<0.001
**EGDS performed**	13 (2.0)	34 (37.0)	<0.001
**EDLOS, minutes**			
Median, IQR	37.0 [26.0–58.0]	74.0 [37.0–184.5]	<0.001

* Values are presented as number (%) unless otherwise indicated. Age and foreign body size are expressed as mean ± standard deviation (SD) and median [interquartile range, IQR]. *p*-values were calculated using the chi-square test, Fisher’s exact test, or Mann–Whitney U test, as appropriate. EGDS = esophagogastroduodenoscopy; EDLOS = emergency department length of stay.

**Table 4:** Multivariable logistic regression for predictors of EGDS.

*	Adjusted Odd Ratio	95% CI	*p*-Value
**Age group**			
Infant (Reference)			
Toddler	1.85	0.22–15.35	0.571
Preschooler	0.96	0.10–8.91	0.971
School—aged	0.17	0.01–2.85	0.221
**Male**	0.78	0.19–3.15	0.722
**Foreign body type**			
Battery (Reference)			
Coin	7.26	1.07–49.31	0.042
Magnetic	1.44	0.19–11.03	0.723
**Symptom present**	1.34	0.31–5.81	0.700
**High risk**	179.36	29.90–1075.78	<0.001

* Values are presented as adjusted odds ratio (aOR) with 95% confidence interval (CI). Infant age group, battery ingestion, and absence of symptoms were used as reference categories. High-risk ingestion was defined as button battery, multiple magnets, sharp objects, or foreign bodies ≥ 2.5 cm in diameter.

## Data Availability

Data is unavailable due to privacy.

## Appendix A

**Table A1:** High-risk foreign body criteria.

High-Risk Foreign Body Criteria
sharp or wide/elongated objects (≥2.5 cm in width or ≥6 cm in length)
multiple FBs
button batteries
evidence of perforation
a coin at the cricopharyngeus muscle level
airway compromise
presence ≥ 24 h
